# Cellular Cannibalism: Suggesting a Histopathological Parameter for Predicting Metastasis of Osteosarcoma in Future Studies

**DOI:** 10.31557/APJCP.2021.22.7.1985

**Published:** 2021-07

**Authors:** Massoumeh Zargaran

**Affiliations:** *Department of Oral and Maxillofacial Pathology, Faculty of Dentistry, Kurdistan University of Medical Sciences, Sanandaj, Iran. *

**Dear Editor**

I read with great interest the article of Chua et al., (2021) entitled “*Evaluation of the receptor activator of nuclear factor kappa B ligand (RANKL) expression in osteosarcoma and its association with the clinicopathological data*”. Osteosarcoma (OS) is the most common primary malignant tumor in children and young adults, associated with high morbidity. This tumor is locally aggressive and has a high tendency to metastasize, especially in the lungs. Despite recent advances in the OS treatment, resulting in a significant increase in the patients’ survival rate, the survival of patients with lung metastasis remains poor. Prediction of early metastasis by identifying predictive markers can lead to appropriate treatment decisions, tailored to the patient’s condition (Ren et al., 2016; Mardani et al., 2020). Although several markers have been suggested in the literature (Ren et al., 2016; Mardani et al., 2020), most of the used techniques are expensive and require procedures that are not available to all patients. Therefore, it is important to find a parameter that is easily recognizable via H&E staining, without the need for advanced diagnostic modalities.

**Figure 1:**
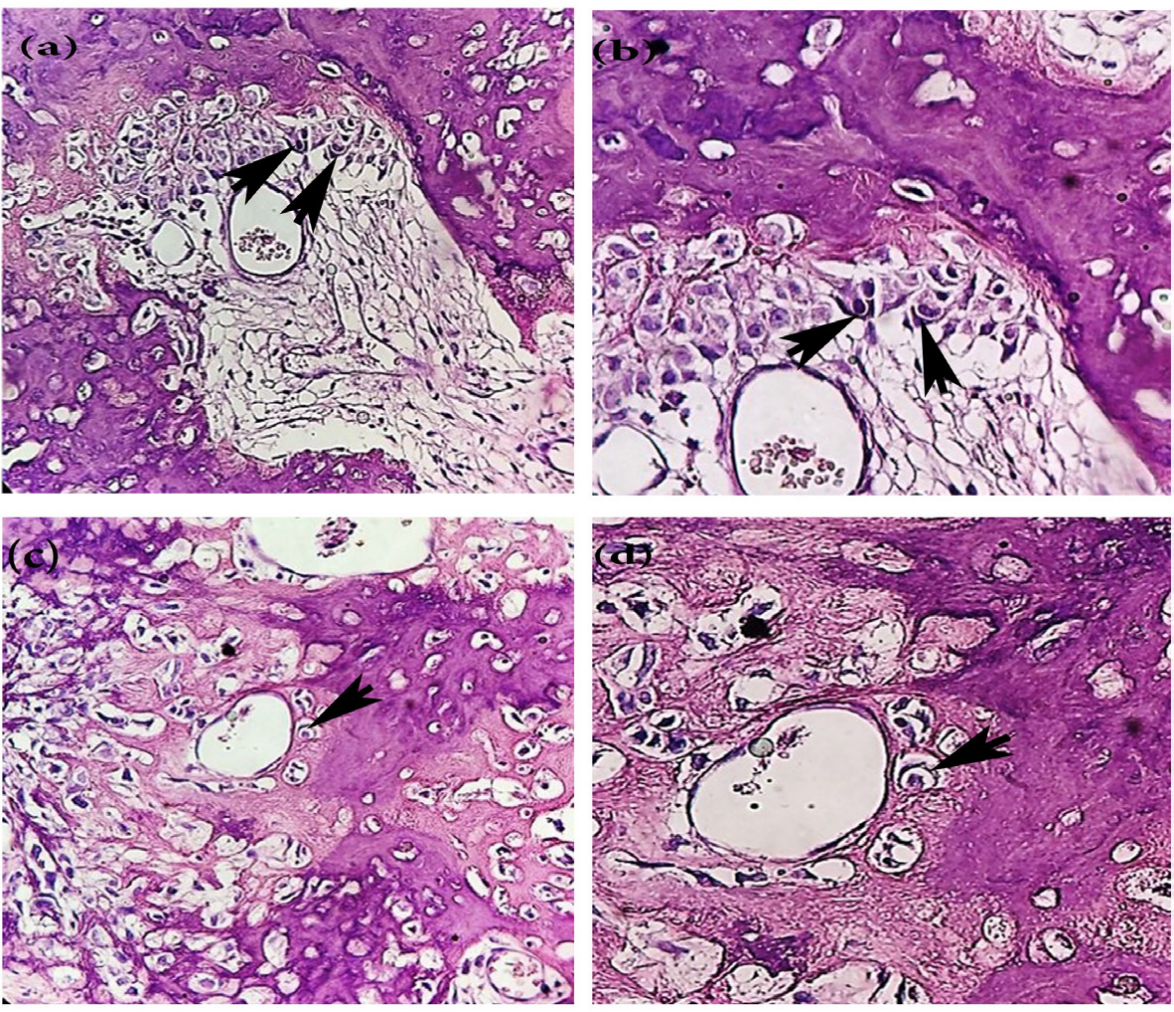
Histopathologic Feature of Osteosarcoma and Cellular Cannibalism. a and c show malignant mesenchymal cells intermixed with disorganized bone and a number of cannibalistic cells( H&E stain, ×100). The black arrows show incomplete cellular cannibalism which a tumor cell is partially enclosed another tumor cell (b, H&E stain, ×400). The black arrow shows a complete cellular cannibalism phenomenon (d, H&E stain, ×400)

Cellular cannibalism (CC) refers to the ability of one cell to engulf another living cell, eventually leading to the death of the internalized cell (Jain, 2015). From a microscopic perspective, CC is seen as a cell with a crescent-shaped nucleus, which partially or completely engulfs another cell with a round to oval nucleus (Jose et al., 2014; Jain, 2015). The cause of CC in malignant tumors seems to be the elimination of nutritional defects in tumor cells and maintaining their survival, but this phenomenon indirectly represents the unfavorable tumor microenvironment, such as hypoxia and acidity (Jain, 2015). The microenvironment acidity plays an essential role in the potent activity of proteolytic enzymes involved in tumor invasion and metastasis and the selection of cell colonies capable of surviving at low pH (cannibalistic cells), and on the other hand, it is a constant feature of metastatic tumor cells (Fais, 2007). The study of CC and its cellular grade in several malignant tumors, such as squamous cell carcinoma (SCC), has shown that this phenomenon is associated with the aggressiveness and metastasis of cancer, especially in advanced grades, and can be a reliable predictor of the metastatic potential of malignant tumor cells (Jose et al., 2014). CC has not been reported in OS yet, which is possibly due to failure to carefully observe this phenomenon rather than its absence in routine histopathological analyses. In other words, we can identify CC in some of OSs by increasing our diagnostic precision (Figure 1) and suggest the possibility of a link between CC and OS metastasis. If this hypothesis is approved in future studies, this parameter may be used to identify patients with a high risk of OS metastasis. Therefore, management and selection of more adaptable treatment plans, depending on the patient’s condition, can decrease the likelihood of metastasis and improve the survival rate of patients.
